# Seasonal pattern of peptic ulcer hospitalizations: analysis of the hospital discharge data of the Emilia-Romagna region of Italy

**DOI:** 10.1186/1471-230X-10-37

**Published:** 2010-04-15

**Authors:** Roberto Manfredini, Roberto De Giorgio, Michael H Smolensky, Benedetta Boari, Raffaella Salmi, Davide Fabbri, Edgardo Contato, Mauro Serra, Giovanni Barbara, Vincenzo Stanghellini, Roberto Corinaldesi, Massimo Gallerani

**Affiliations:** 1 Department of Internal Medicine, Hospital of the Delta, Lagosanto, Azienda Unità Sanitaria Locale, Ferrara, Italy; 2 Department of Clinical and Experimental Medicine, Section Clinica Medica, University of Ferrara, Italy; 3 Department of Clinical Medicine, University of Bologna, Italy; 4 Department of Biomedical Engineering, the University of Texas at Austin, USA; 5 Department of Internal Medicine, Azienda Ospedaliera-Universitaria S. Anna, Ferrara, Italy; 6 Medical Direction, Azienda Ospedaliera-Universitaria S. Anna, Ferrara, Italy; 7 Medical Direction, Azienda Unità Sanitaria Locale, Ferrara, Italy

## Abstract

**Background:**

Previous studies have reported seasonal variation in peptic ulcer disease (PUD), but few large-scale, population-based studies have been conducted.

**Methods:**

To verify whether a seasonal variation in cases of PUD (either compicated or not complicated) requiring acute hospitalization exists, we assessed the database of hospital admissions of the region Emilia Romagna (RER), Italy, obtained from the Center for Health Statistics, between January 1998 and December 2005. Admissions were categorized by sex, age (<65, 65-74, ≥ 75 yrs), site of PUD lesion (stomach or duodenum), main complication (hemorrhage or perforation), and final outcome (intended as fatal outcome: in-hospital death; nonfatal outcome: patient discharged alive). Temporal patterns in PUD admissions were assessed in two ways, considering a) total counts per single month and season, and b) prevalence proportion, such as the monthly prevalence of PUD admissions divided by the monthly prevalence of total hospital admissions, to assess if the temporal patterns in the raw data might be the consequence of seasonal and annual variations in hospital admissions *per se *in the region. For statistical analysis, the χ2 test for goodness of fit and inferential chronobiologic method (Cosinor and partial Fourier series) were used.

**Results:**

Of the total sample of PUD patients (26,848 [16,795 males, age 65 ± 16 yrs; 10,053 females, age 72 ± 15 yrs, *p *< 0.001)], 7,151 were <65 yrs of age, 8,849 between 65 and 74 yrs of age, and 10,848 ≥ 75 yrs of age. There were more cases of duodenal (DU). (89.8%) than gastric ulcer (GU) (3.6%), and there were 1,290 (4.8%) fatal events. Data by season showed a statistically difference with the lowest proportion of PUD hospital admissions in summer (23.3%) (*p *< 0.001), for total cases and rather all subgroups. Chronobiological analysis identified three major peaks of PUD hospitalizations (September-October, January-February, and April-May) for the whole sample (*p *= 0.035), and several subgroups, with nadir in July. Finally, analysis of the monthly prevalence proportions yielded a significant (p = 0.025) biphasic pattern with a main peak in August-September-October, and a secondary one in January-February.

**Conclusions:**

A seasonal variation in PUD hospitalization, characterized by three peaks of higher incidence (Autumn, Winter, and Spring) is observed. When data corrected by monthly admission proportions are analyzed, late summer-autumn and winter are confirmed as higher risk periods. The underlying pathophysiologic mechanisms are unknown, and need further studies. In subjects at higher risk, certain periods of the year could deserve an appropriate pharmacological protection to reduce the risk of PUD hospitalization.

## Background

Peptic ulcer disease (PUD) is representative of a group of ulcerative disorders of the upper gastrointestinal tract (GIT), mainly involving the stomach and duodenum, that share a common acid-pepsin pathogenesis [1]. The major PUD are duodenal (DU) and gastric ulcer (GU). A wide array of medical conditions exhibits seasonal patterns in their occurrence. Winter months, for example, exhibit a higher frequency of unfavourable cardiovascular events, such as acute myocardial infarction and sudden cardiac death [2-4], aortic rupture or dissection [5,6], stroke [7], and venous thromboembolism [8,9]. Although a possible seasonality for PUD has been extensively investigated (Table 1), there is no consensus as to the peak season(s) of greatest acute disease activity. PUD is not a single entity, as it may be categorized according to the lesion site, i.e., stomach or duodenum, and presence or absence of complications, i.e., hemorrhage or perforation. Some studies have focused on PUD, or on GU or DU, and others on complicated ulcers, while several studies have the limitation to be based on small sample size populations. Based on the literature, we hypothesized that hospital admissions for acute PUD would show seasonality, as would the associated complications and fatal outcomes. Thus, the present investigation explores the annual pattern in severe acute PUD requiring hospitalization, in relation to patient's sex, age, type of lesion (GU or DU), disease complications, and (fatal/nonfatal) outcome using the large-scale hospital discharge database maintained for the Emilia Romagna Region (RER) of Italy.

**Table 1:** Seasonal variation in the onset of PUD and related complications in several countries and continents.

Disease	Author/Setting	Sample size	Source of data	Time period	Peak
**Peptic ulcer**					
	Scholtyssek et al, 1986 (Germany)	1091	Single center	1973 - 1983	Autumn (Nov)
	Sonnenberg et al 1992 (U.S.A.)	Unknown	Nationwide database	Not available	Jan to Mar, Oct
	Savarino et al, 1996 (Italy)	319	Single center	1987 - 1992	Autumn (Nov-Dec) Winter (Jan-Mar)

**Hemorrhage**					
	Marbella et al, 1988 (U.S.A.)	285	Single center	1974 - 1976	Jan-Feb, Jul-Aug, Nov-Dec
	Tishchenko et al, 1990 (Russia)	390	Single center	Not available	Jan, Oct, Sept
	Shih et al, 1993 (Taiwan)	2,889	Single center	1987 - 1992	Jan - Feb
	Thomopoulos et al, 1997 (Greece)	1,992	Single center	1991 - 1996	Apr and Oct
	Tsai et al, 1998 (Taiwan)	10,331	Single center	1989 - 1996	Nov - Mar
	Rodrìguez et al, 1999 (Mexico)	275	Single center	1991 - 1997	May, June, Nov
	Nomura et al, 2001 (Japan)	441	Single center	1996 - 1999	Autumn and Winter
	Lopez-Cepero et al, 2005 (Spain)	499	Single center	1998 - 2001	No seasonal variation

**Perforation**					
	Adler et al, 1984 (Australia)	1,187	Hospitals of West. Australia	1971 - 1981	Nov - Jan
	Christensen et al, 1988 (Denmark)	296	7 depts of gastrointest. surg.	1975 - 1984	August-September
	Bendahan et al, 1992 (Israel)	540	Single center	1977 - 1986	Nov - Feb
	Csendes et al, 1995 (Chile)	Unknown	9 hospitals	1980,1985,1990	Autumn
	Yen et al, 1996 (Taiwan)	1,787	Single center	1991 - 1992	Feb-Mar
	Svanes et al, 1998 (Norway)	1,480	Area hospitals	1935 - 1990	May-Jul, Nov-Dec
	Wysocki et al, 1999 (Poland)	Unknown	Single center	1991 - 1995	May-Jul, Sep-Oct
	Janik & hwirot, 2000 (Poland)	441	Single center	1977 - 1996	No seasonal variation
	Budzynski et al, 2000 (Poland)	220	Single center	1993 - 1997	No seasonal variation
	Liu et al, 2003 (China)	24,252	17 hospitals	1992 - 1997	Winter and Spring
	Kocer et al, 2006 (Turkey)	269	Single center	2001 - 2004	Winter

## Methods

The study was conducted with the approval of the local institutional committees for human research. The analysis included all consecutive hospital admissions for PUD that occurred between January 1, 1998 and December 31, 2005, as recorded in the database of the RER Center for Health Statistics. The RER is situated in north-eastern Italy and has a surface area of 22,124 Km^2^, has a total population of ~3,985,000 people (≈ 7% of the total population of Italy), with a density of 180 persons/Km^2^.

### Data collection

Starting from 1998, the RER elaborated an electronic database tracking all discharge hospital sheets (DHS - or "SDO" in Italian) of persons admitted to public and private hospitals, which collected an overall total of ~5.6 million cases as of the latest updated database (December 2005) used for this investigation. The DHS lists the name, sex, date of birth, date of hospital admission and discharge, department of admission and discharge, up to nine discharge diagnoses, and the most important diagnostic procedures coded according to the *International Classification of Diseases*, 9th Revision, Clinical Modification (ICD-9-CM). The RER health authorities removed patient name, exact address, and other potential identifiers from the database provided for this study, to respect national dispositions-by-law in terms of privacy. Birth date was the only identification data allowed for analysis, in order to categorize the admissions by age group and to clean the database of potential repeat hospital admissions of the same person. We sought to include in the statistical analyses only the calendar date of the first hospital admission of any given PUD crisis per patient, to adhere to the statistical assumption of independency of observations. It is possible, however, that data of different (multiple) patients with a same birth date could have been inappropriately deleted because they were believed to be a readmission for an ongoing PUD crisis of the same person, but the occurrence of such cases was rare, with no potential impact on the findings, given the almost 27,000 total PUD admissions considered. Of course, since the electronic RER database started on 1998, no information is available on cases of PUD admissions prior to that year.

The unit for the statistical analysis of the data was admission date for acute PUD as per ICD-9-CM codes 531.00 to 534.91. Only admissions directly related to PUD, i.e., only cases in which PUD was indicated as the main discharge diagnosis, were abstracted from the database. The total sample of hospital admission data was divided into subgroups categorized by sex, age (<65, 65-74, >75 yrs), site of lesion -- gastric ulcer or GU (ICD-9-CM codes from 531.00 to 531.91), duodenal ulcer or DU (ICD-9-CM codes from 532.00 to 532.91), peptic ulcer (PU), site unspecified (ICD-9-CM codes from 533.00 to 533.91), and gastrojejunal ulcer (ICD-9-CM codes from 534.00 to 534.91). They were also categorized by main complication, i.e., hemorrhage (ICD-9-CM codes due to UG: 531.0, 531.2, 531.4, 531.6; due to DU 532.0, 532.2, 532.4, 532.6; due to PU site unspecified 533.0, 533.2, 533.4, 533.6; due to gastrojejunal ulcer 534.0, 534.2, 534.4, 534.6), perforation (ICD-9-CM codes due to UG: 531.1, 531.2, 531.5, 531.6; due to DU 532.1, 532.2, 532.5, 532.6; due to PU site unspecified 533.1, 533.2, 533.5, 533.6; due to gastrojejunal ulcer 534.1, 534.2, 534.5, 534.6), and a rough indicator of outcome, i.e., fatal: in-hospital death, and nonfatal: patient discharged alive.

### Statistical analysis

We used SPSS 13.0 for Windows, SPSS Inc., Chicago, IL, 2004 for statistical analysis of demographic data, e.g., age comparisons between men and women.

Temporal patterns in PUD admissions were assessed in two ways, considering a) total counts per single month and season (Spring: March 21-June 20, Summer: June 21-September 22, Fall: September 23-December 20; Winter: December 21-March 20), and b) prevalence proportion, such as the monthly prevalence of PUD admissions divided by the monthly prevalence of total hospital admissions, to assess if the temporal patterns in the raw data might be the consequence of seasonal and annual variations in hospital admissions *per se *in the region.

The distribution of the total and subgroup PUD hospital admissions within the four 3-month periods (seasons) of the year was tested for uniformity by the χ2 test for goodness of fit. Analysis of annual variation in admissions was performed by applying a partial Fourier series to the time series data using an internationally validated method (Chronolab software on an Apple Macintosh computer) [10]. This method selects the harmonic, or combination of harmonics (cosine waveforms), that best explains the temporal variance of the data. The percentage of the overall variance attributable to the approximated cosine function serves as the estimate of the goodness of fit, with the F-test statistic applied to the variance accounted by the single or multiple cosine curve approximation versus straight-line approximation of the time series data to accomplish a test of the null hypothesis of zero amplitude, i.e., absence of significant temporal variation for the given period of the approximated curve function. The parameters calculated for the overall 1 yr in period (τ) cosine approximation of the time series data (τ = 8766 h) were: the midline estimated statistic of rhythm (MESOR, the rhythm-adjusted mean for the time period analyzed), amplitude (half the difference between the absolute maximum and minimum of the fitted approximation), and peak (acrophase) and trough (bathyphase) time referenced to 00:00 h December 31. Significance levels were set at *p *< 0.05.

## Results

Between January 1, 1998 and December 31, 2005, the database of the RER identified 26,848 hospital admissions corresponding to ICD-9-CM codes indicative of PUD; 16,795 (62.5%) were males (mean ± SD) 65 ± 16 yrs of age and 10,053 (37.5%) females 72 ± 15 yrs of age (t = 35,508 *p *< 0.001). PUD admissions were more commonly associated with advanced age; 7,151 were <65 yrs of age, 8,849 between 65 and 74 yrs of age, and 10,848 >75 yrs of age. There were more cases of duodenal (DU). (89.8%) than gastric ulcer (GU) (3.6%). As for outcome, there were 1,290 (4.8%) admissions culminated in an in-hospital death. Overall, 15,223 cases of complications were associated with complications, 12,682 being hemorrhage (47.2%) and 2,541 (9.5%) perforation.

### Conventional analysis

**Figure 1:**
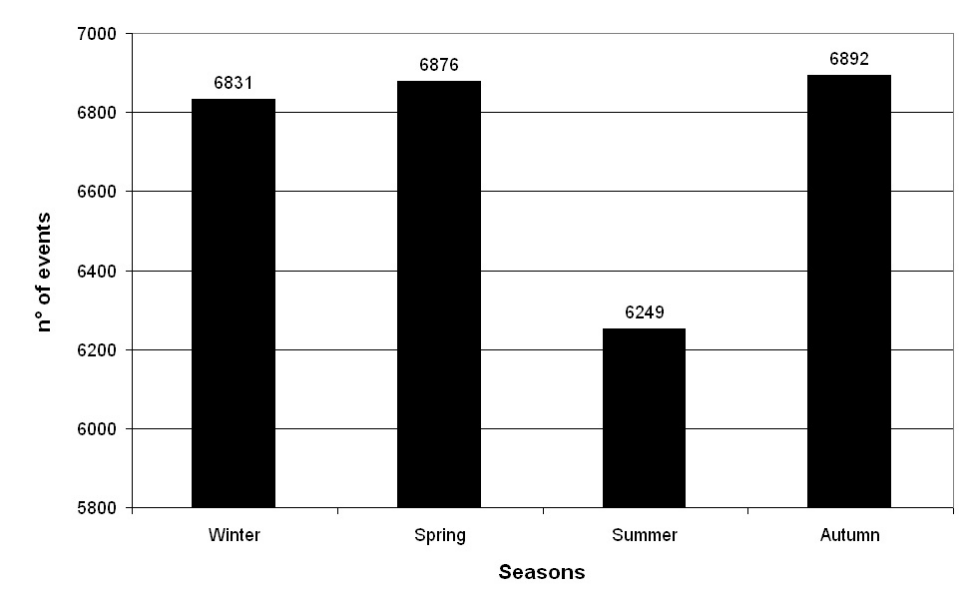
**Seasonal distribution of severe PUD hospitalizations in the Emilia Romagna Region of Italy**.

**Figure 2:**
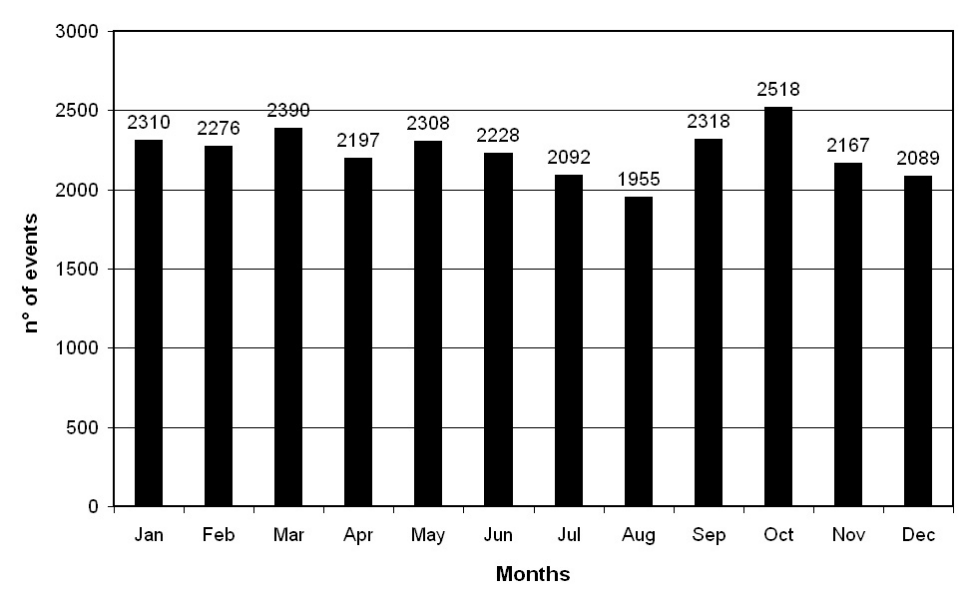
**Monthly distribution of severe PUD hospitalizations in the Emilia Romagna Region of Italy**.

When the raw data were categorized by season (Figure 1), hospital admissions involving the PUD codes were least frequent (6,249 or 23.2%) in summer (goodness of fit χ2 = 42.88, p < 0.001). This findings was similar for admissions categorized by sex, age, and final outcome (fatal/non-fatal) (Table 2). Figure 2 presents in greater detail the number of admissions is fewest in August (1,955 or 7.28%) and highest in autumn, particularly in October (2,518 or 9.37%) - the absolute peak, and that they are also elevated in the months of January (2,310 or 8.6%), March (2,390 or 8.9%), and May (2,308 or 8.6%).

**Table 2:** Seasonal distribution of peptic ulcer: χ2 test for goodness of fit.

		n°	winter n°(%)	spring n°(%)	summer n°(%)	autumn n°(%)	Χ2	p	Difference between subgroups	
**Total of sample**		26,848	6,831 (25.4)	6,876 (25.6)	6,249 (23.3)	6,892 (25.7)	42.88	<0.001		

**Gender**	***Women***	10,053	2,483 (24.7)	2,656 (26.4)	2,381 (23.7)	2,533 (25.2)	15.59	0.002	t = 10.10	p = 0.022
	***Men***	16,795	4,348 (25.9)	4,220 (25.1)	3,868 (23.0)	4,359 (26.0)	37.58	<0.001		

**Age subgroups**	***<65 yrs***	7,151	1,754 (24.5)	1,807 (25.3)	1,680 (23.5)	1,910 (26.7)	15.71	0.001	t = 16.62	p = 0.011
	***65-74 yrs***	8,849	2,358 (26.6)	2,208 (25.0)	2,047 (23.1)	2,236 (25.3)	22.21	<0.001		
	***≥ 75 yrs***	10,848	2,719 (25.1)	2,861 (26.4)	2,522 (23.2)	2,746 (25.3)	21.94	<0.001		

**Final outcome**	***Fatal***	1,290	358 (27.8)	326 (25.3)	317 (24.6)	289 (22.4)	7.52	n.s.	t = 9.51	p = 0.03
	***Non fatal***	25,558	6,473 (25.3)	6,550 (25.6)	5,932 (23.2)	6,603 (25.8)	45.01	<0.001		

**With hemorrhage**		12,682	3,247 (25.6)	3,172 (25.0)	2,970 (23.4)	3,293 (26.0)	19.26	<0.001		

**With perforation**		2,541	596 (23.5)	688 (27.1)	593 (23.3)	664 (26.1)	10.92	0.013		

**Gastric ulcer**	***Total***	976	242 (24.8)	242 (24.8)	212 (21.7)	280 (28.7)	9.54	0.024		
	***Acute***	734	181 (24.7)	179 (24.4)	164 (22.3)	210 (28.6)	6.07	n.s.	t = 0.758	p = n.s.
	***Chronic***	242	61 (25.2)	63 (26.0)	48 (19.8)	70 (28.9)	4.18	n.s.		

**Duodenal ulcer**	***Total***	24,105	6,075 (25.2)	6,180 (25.6)	5,636 (23.4)	6,214 (25.8)	35.44	<0.001		
	***Acute***	12,180	3,038 (24.9)	3,136 (25.7)	2,866 (23.5)	3,140 (25.8)	16.22	0.001	t = 30.37	p < 0.001
	***Chronic***	7,415	1,594 (21.5)	2,004 (27.0)	1,814 (24.5)	2,003 (27.0)	61.47	<0.001		

**Peptic ulcer, site unspecified**	***Total***	2,795	768 (27.5)	711 (25.4)	622 (22.3)	694 (24.8)	15.56	0.002		
	***Acute***	359	91 (25.3)	85 (23.7)	86 (24.0)	97 (27.0)	1.04	n.s.	t = 1.788	p = n.s.
	***Chronic***	2,383	628 (26.4)	626 (26.3)	536 (22.5)	593 (24.9)	9.3	0.026		

**Gastrojejunal ulcer**		51	17 (33.3)	13 (25.5)	10 (19.6)	11 (21.6)	2.25	n.s.		

Statistical analysis: χ2 test for goodness of fit (SPPS 13.0 for Windows, Chicago, IL, 2004).

### Inferential analysis

Chronobiological analysis involving the approximation of the fundamental 1 yr and up to three additional harmonics identified a significant annual pattern in PUD characterized by three main peaks (September-October, January-February, and April-May) for the entire sample of admissions (*p *= 0.035). The findings of this objective inferential time series analysis are representative of the raw data shown in Figure 2. A statistically significant annual pattern was also verified for the data of the following: females (*p *= 0.032), 65-74 yrs of age (*p *= 0.022), GU (*p *= 0.002), and DU (*p *= 0.036). With the exception of a few subgroups, the nadir of admissions consistently occurred in the month of July (Table 3).

**Table 3:** Seasonal distribution of PUD hospital admission: results of chronobiological time series analysis.

		n	PR	MESOR (± SE)	Peak (1°, 2°, 3°)	Nadir	P*
**Total cases**		26,848	83.3	2,236 (26.38)	Sept/oct, Jan/Feb, Apr/May	July	0.035

**Males**		16,795	78.0	1,398.69 (20.43)	Sept/Oct, Jan/Feb, Apr/May	July	0.064

**Females**		10,053	87.1	837.43 (9.24)	Sept/Oct, Apr/May, Jan/Feb	July	0.032

**Age < 65 yrs**		7,151	73.8	595.77 (8.89)	Sept/Oct, Jan/Feb, Apr/May	July	0.144

**Age 65-74 yrs**		8,849	86.8	736.75 (9.60)	Jan/Feb, Sept/Oct, Apr/May	July	0.022

**Age > 75 yrs**		10,848	71.8	903.60 (14.80)	Sept/Oct, Apr/May, Jan/Feb	July	0.123

**Final outcome: fatal**		1,290	58.7	107.48 (3.03)	Dec/Jan, Apr/May, Aug/Sept	October	0.407

**Final outcome: non-fatal**		25,558	83.6	2,128.64 (25.71)	Sept/Oct, Jan/Feb, Apr/May	July	0.067

**With hemorrhage**		12,682	63.3	1,056.51 (18.37)	Sept/Oct, Jan/Feb, Apr/May	July	0.153

**With perforation**		2,541	29.0	211.73 (7.62)	Mar/May, Sept/Nov, -	July	0.949

**Gastric ulcer**	***Total***	976	96.3	81.25 (0.83)	Sept/Oct, Jan/Feb, Apr/May	July	0.002
	***Acute***	734	88.7	61.11 (1.04)	Sept/Oct, Jan/Feb, Apr/May	July	0.021
	***Chronic***	242	75.6	20.13 (0.80)	Sept/Oct, Jan/Feb, Apr/May	July	0.161

**Duodenal ulcer**	***Total***	24,105	82.7	2,007.72 (24.40)	Sept/Oct, Jan/Feb, Apr/May	July	0.036
	***Acute***	12,180	95.1	1,014.62 (6.21)	Sept/Oct, Apr/May, Jan/Feb	July	0.002
	***Chronic***	7,415	73.5	617.98 (17.35)	Sept/Nov, Apr/Jun, -	July	0.083

*P value for the test of null-amplitude hypothesis; rejection of the hypothesis at *p *< .05 the amplitude of the annual pattern = 0 constitutes evidence for annual variation.

**Figure 3:**
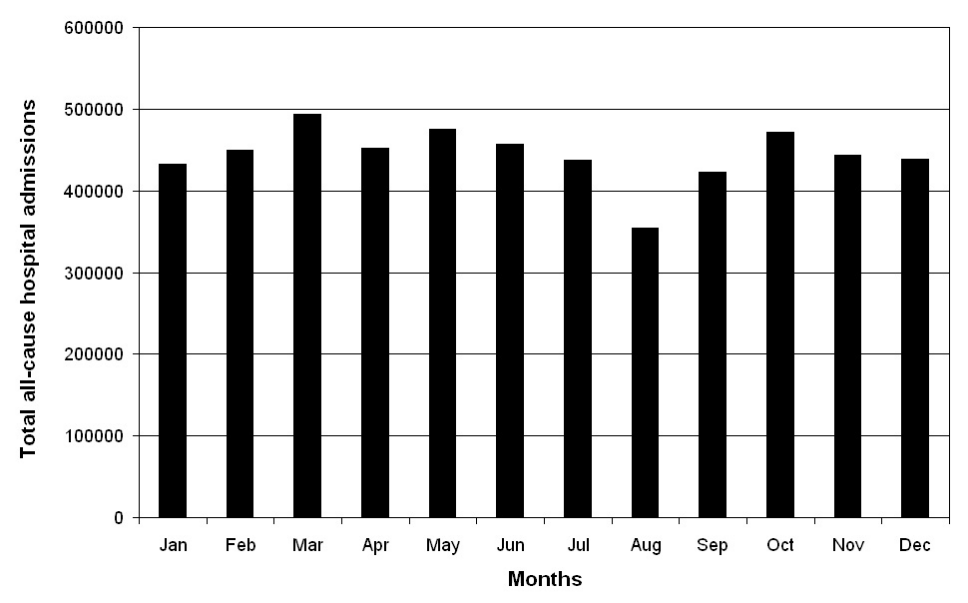
**Total all-cause hospital admissions by month of the year (1998-2005) in the Emilia Romagna Region of Italy**.

**Figure 4:**
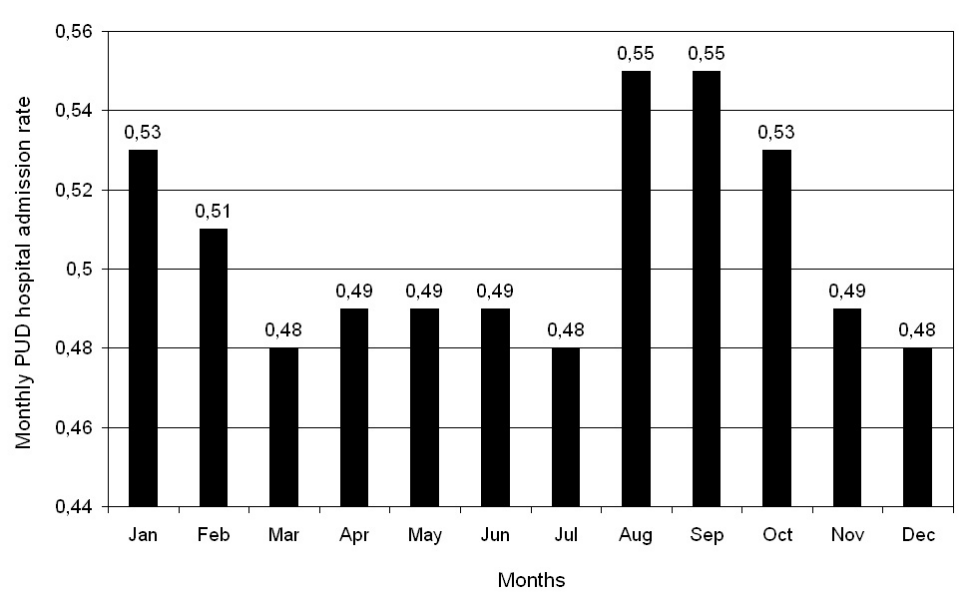
**Monthly PUD hospital admission rate (number of PUD admissions relative to total number of all hospital admission, independent of cause each month) in the Emilia Romagna Region of Italy**.

The PUD data were also analyzed in terms of monthly prevalence proportion, i.e., the monthly prevalence of PUD admissions divided by the monthly prevalence of total admissions. Figure 3 shows the pattern in the all-cause monthly admissions in the RER. Total admissions were highest in the spring months of March and May and also the autumn months of October, November, and December. They are lowest, considerably so, in August. Cosinor analysis did not reveal a significant seasonal pattern for total RER admissions (*p *= 0.095). The seasonal variation in total hospital admissions, i.e., between the spring peak and summer trough, amounts to ~13%. Figure 4 shows the temporal pattern of the adjusted rates of PUD admissions differs from the raw number of PUD admissions depicted in Figure 2. Figure 4 depicts a two-peak pattern in PUD hospital admission rates, with a major peak in the very late summer-early autumn encompassing the months of August through October, and a second less prominent peak in winter encompassing the months of January and February. Statistically significant seasonal differences in PUD hospital admission proportions are also documented. Admission proportions are greatest in winter and lowest in spring. The PUD rate by season is: Winter 6,831/1,321,476 or 5.17 per 1,000 admissions, Spring 6,876/1,420,857 or 4.84 per 1000 admissions, Summer 6,249/1,249,034 or 5.00 per 1,000 admissions, Autumn 6,892/1,338,461 or 5.15 per 1,000 admissions (goodness of fit χ2 = 19,142, *p *< 0.0001). Inferential analysis of the PUD hospital admission rate data, exploring the fundamental 1 yr and up to three best fitting harmonics, reveals a statistically significant (*p *= 0.025), modest-amplitude (total peak-trough variation equal to 16% of the MESOR) annual variation with the major peak in August-September and secondary peak in January-February. The findings of the objective inferential analysis are representative of the monthly rate data shown in Figure 4.

## Discussion

Peptic ulcerations of the gastro-duodenal tract are defects of the mucosal lining resulting from the epithelial cell damaged evoked by acid and pepsin as caustic agents [11]. From the pathophysiological standpoint, PUD can be viewed as the consequence of the noxious effects of aggressive factors (i.e., acid and pepsin) prevailing over the defensive mechanisms of the gastroduodenal mucosa [12]. According to the old, though ever valid, Schwartz's aphorism ("no acid, no ulcer"), gastric acid is of paramount importance among the aggressive factors in PUD. However, the discovery of *Helicobacter pylori *changed the perspective that PUD is simply a disorder of gastric acid homeostasis. Considerable research has clearly established that *H. pylori *is a significant cause of gastritis and PUD. Indeed, acid hypersecretion, which is commonly observed in PUD, is more likely the result of *H. pylori *infection rather than being the primary abnormality [13]. Recent epidemiological surveys showed a decrease in the incidence of all upper gastrointestinal bleeding, but the incidence of PU bleeding (that is responsible for about 50% of all cases), has remained stable [14]. Peptic ulceration and complications, such as perforation and bleeding, also result from the use of non-steroidal anti-inflammatory drugs (NSAIDs) [12,15]. Based on experimental research and studies in humans, the GIT has been shown to have a specific temporal organization, which shows circadian rhythms in several parameters including gastric pH, digestive enzymes, mucosal cell replication, and blood flow [16,17]. Several human studies [18-22] also suggest circadian variation in gastrointestinal bleeding. Annual or seasonal variation in the chronophysiology and balance of gastro-aggressive and gastro-defensive processes of the GIT has yet to be explored, whereas annual patterns in acute GIT conditions have often been reported.

Although seasonal variation in PUD onset has been rather extensively investigated, the conclusions of the previous studies, with the exclusion of two large investigations performed in the USA [23] and in China [24], are limited primarily because of small sample size. The present study, which is based on 27,000 hospital admissions, reveals an annual pattern in the hospital admission of severe PUD independent of patient sex, age, type of lesion (GU or DU), main complication (hemorrhage or perforation), and final outcome (fatal vs. non-fatal). We analyzed the PUD admission data in two different ways, i.e. as raw admission counts/month or season and also as prevalence proportions, i.e. number of PUD admissions versus number of all admissions independent of medical reasons/month and season. The former approach revealed several peaks in PUD hospital admissions during the year, with the most prominent one in October and lesser ones in January, March, and May. The latter approach suggested a 6-month cycle, with the major peak in admission rates in the very late summer (August-September) and secondary peak in January-February.

Seasonal variation in PUD, either complicated or uncomplicated, and typically investigated as the number of cases (rather than prevalence proportions) per month or season, has been a common research topic over the years, (Table 1). Autumnal and winter peaks have been reported in Germany [25], USA [23], and Italy [26]. The most frequent complications of PUD were hemorrhage and perforation. As for hemorrhage, autumn or winter peaks have been found again in the USA [27], Russia [28], Taiwan [29,30], and Japan [31]. Thomopoulos et al. [32] in Greece, Yen et al. [33] in Taiwan, and Rodriguez et al in Mexico [34] found summer/autumn peaks, while Lopez-Cepero et al. [35] in Spain found no seasonality, and in Norway Svanes et al. [36] reported a 6-month time pattern. A different seasonal variation, with spring-summer peaks, was reported in Australia [37], Denmark [38], and Poland [39]. On the other hand, winter peaks have been observed in Turkey [40], Israel [41], and China [24], and autumn peak in Chile [42]. Other studies in Poland [43,44], however, found no evidence of seasonality.

Why PUD is more frequent in winter and late summer-autumn months? Although the answer is far from being conclusive several factors have been claimed to play a role in PUD seasonality. Concerning severe PUD, Nomura et al. [31] found that the incidence of hematemesis due to GU over the year showed an inverse temporal relationship to temperature and relative humidity and a parallel relationship with atmospheric pressure. Liu et al. [24,45] found a close relationship between detectable PUD and average temperature, average highest and lowest temperature, average atmospheric pressure, and the average dew point temperature. A recent nation-wide study conducted in Taiwan by Xirasagar et al. [46] found DU admissions were negatively associated with temperature, with a winter peak and summer trough in patients 35-49 and >50 yrs of age. On the other hand, a Spanish study [35] failed to find a correlation between the incidence of GU bleeding and ambient temperature, atmospheric pressure, relative humidity, wind direction and speed.

Seasonality of *H. pylori *infection is another possible factor which may affect PUD onset. Savarino et al. [26], however, did not find any difference in the percentage of *H. pylori*-positive DU cases between seasons or a parallel annual fluctuation in gastric acidity and *H. pylori *infection. These findings are in line with another study showing no significant correlation between the seasonal differences in the diagnosis of ulcer disease and presence of *H. pylori *infection [47]. Whether NSAIDs, which are known to damage the epithelium of the GIT, play a role in the seasonal pattern of PUD hospitalizations remains unsettled. Circadian rhythms in the tolerance of the GIT to NSAIDs have been demonstrated in clinical studies. Lévi et al. [48] found that indomethacin taken once-a-day in the morning makes GIT mucosa more prone to damage than evening intake of the drug. Similar findings were obtained by Perpoint et al. [49] for ketoprofen. Both these clinical studies are consistent with the report of Moore & Goo [50] showing that a single high (1 gram) dose of aspirin in the morning (10:00 h) produced twice the number of gastric lesions than did evening (22:00) ingestion. We are unaware of any studies that have addressed annual or seasonal variation in the tolerance of the GIT to NSAIDs in humans. In laboratory animal studies, Leng [51] found that the ulcerogenic effect (maximal area of ulceration) of phenylbutazone in rats was greatest in October and December, while for acetylsalicylic acid it was February and March. It is theoretically possible that low temperature of these months could exacerbate co-morbidities, such as rheumatoid or osteoarthritis, thereby motivating greater reliance on corticosteroids and/or non-steroid anti-inflammatory medications.

Alcohol is another powerful gastrolesive variable in ulcerogenesis, and it is plausible that seasonal differences in alcohol consumption might contribute to seasonality of severe PUD hospital admissions. However, the seasonal pattern in drinking behavior has not been extensively studied. The only indirect available evidence reported a winter to summer increase in per-capita alcohol sales in the U.S.A. [52], and a higher summer frequency of deaths from accidents and incidents associated with alcohol consumption in Moscow [53].

## Conclusions

Some previous studies reported a seasonal pattern for acute PUD, mainly characterized by autumn and winter peaks. The present large-scale, population-based study provides evidence of an annual pattern of PUD hospitalizations in the RER, characterized by peaks in autumn, spring and winter, when raw data were considered. However, when the analysis was done on the prevalence proportions, such as the monthly prevalence of PUD admissions divided by the monthly prevalence of total hospital admissions, a biphasic pattern was shown, with a main peak in late summer-autumn (August-September-October) and a second one in winter (January-February).

We are aware of the limitations of the present work, including lack of data on: 1) weather variables of the RER (characterized by a large extension and wide orographic and climatic conditions); 2) regional (or national) seasonal variation in the severity of patient co-morbidities (i.e., rheumatic diseases and related reliance upon NSAIDs); 3) alcohol consumption. It is possible, in fact, that susceptibility to different factors, such as NSAIDs, alcohol consumption and *H. pylori *infection may have an impact in different seasonal peaks of PUD worsening. Moreover, very little is known about the possible influence of biological rhythms on the occurrence of acute PUD. Although this study does not provide insights into pathogenetic factors, the possible demonstration of seasonal variation in PUD hospital admissions may prompt further research to better understand seasonality and guide appropriate management.

## Competing interests

The authors declare that they have no competing interests.

## Authors' contributions

RM, RDeG, MHS, RC, MG: conceived of the study, participated in the design and coordination of the study, performed the statistical analysis, and participated to draft and critically revise the manuscript. BB, RS, MS, GB, VS: participated in the design of the study, obtained data, participated in the statistical analysis, and participated to draft the manuscript. DF, EC: participated in the design and coordination of the study, helped for obtaining data, and participated to draft the manuscript. All authors read and approved the final manuscript.

## Pre-publication history

The pre-publication history for this paper can be accessed here:

http://www.biomedcentral.com/1471-230X/10/37/prepub
